# The Traumatic Inoculation Process Affects TSPO Radioligand Uptake in Experimental Orthotopic Glioblastoma

**DOI:** 10.3390/biomedicines12010188

**Published:** 2024-01-15

**Authors:** Lukas Gold, Enio Barci, Matthias Brendel, Michael Orth, Jiying Cheng, Sabrina V. Kirchleitner, Laura M. Bartos, Dennis Pötter, Maximilian A. Kirchner, Lena M. Unterrainer, Lena Kaiser, Sibylle Ziegler, Lorraine Weidner, Markus J. Riemenschneider, Marcus Unterrainer, Claus Belka, Joerg-Christian Tonn, Peter Bartenstein, Maximilian Niyazi, Louisa von Baumgarten, Roland E. Kälin, Rainer Glass, Kirsten Lauber, Nathalie L. Albert, Adrien Holzgreve

**Affiliations:** 1 Department of Nuclear Medicine, LMU University Hospital, LMU Munich, Marchioninistr. 15, 81377 Munich, Germany; lukas.gold@med.uni-muenchen.de (L.G.);; 2 Neurosurgical Research, Department of Neurosurgery, LMU University Hospital, LMU Munich, Marchioninistr. 15, 81377 Munich, Germany; 3 Munich Cluster for Systems Neurology (SyNergy), LMU Munich, 81377 Munich, Germany; 4 Department of Radiation Oncology, LMU University Hospital, LMU Munich, Marchioninistr. 15, 81377 Munich, Germany; 5 Department of Radiation Oncology, University Hospital Tübingen, 72076 Tübingen, Germany; 6 Department of Neurosurgery, LMU University Hospital, LMU Munich, Marchioninistr 15, 81377 Munich, Germany; 7 Department of Neuropathology, Regensburg University Hospital, 93053 Regensburg, Germany; 8 DIE RADIOLOGIE, 80331 Munich, Germany; 9 German Cancer Consortium (DKTK), Partner Site Munich, 81377 Munich, Germany; 10 Bavarian Cancer Research Center (BZKF), 81377 Munich, Germany

**Keywords:** TSPO PET and autoradiography, glioblastoma, traumatic brain injury (TBI), orthotopic implantation, immunohistochemistry

## Abstract

Background: The translocator protein (TSPO) has been proven to have great potential as a target for the positron emission tomography (PET) imaging of glioblastoma. However, there is an ongoing debate about the potential various sources of the TSPO PET signal. This work investigates the impact of the inoculation-driven immune response on the PET signal in experimental orthotopic glioblastoma. Methods: Serial [^18^F]GE-180 and *O*-(2-[^18^F]fluoroethyl)-L-tyrosine ([^18^F]FET) PET scans were performed at day 7/8 and day 14/15 after the inoculation of GL261 mouse glioblastoma cells (n = 24) or saline (sham, n = 6) into the right striatum of immunocompetent C57BL/6 mice. An additional n = 25 sham mice underwent [^18^F]GE-180 PET and/or autoradiography (ARG) at days 7, 14, 21, 28, 35, 50 and 90 in order to monitor potential reactive processes that were solely related to the inoculation procedure. In vivo imaging results were directly compared to tissue-based analyses including ARG and immunohistochemistry. Results: We found that the inoculation process represents an immunogenic event, which significantly contributes to TSPO radioligand uptake. [^18^F]GE-180 uptake in GL261-bearing mice surpassed [^18^F]FET uptake both in the extent and the intensity, e.g., mean target-to-background ratio (TBR_mean_) in PET at day 7/8: 1.22 for [^18^F]GE-180 vs. 1.04 for [^18^F]FET, *p* < 0.001. Sham mice showed increased [^18^F]GE-180 uptake at the inoculation channel, which, however, continuously decreased over time (e.g., TBR_mean_ in PET: 1.20 at day 7 vs. 1.09 at day 35, *p* = 0.04). At the inoculation channel, the percentage of TSPO/IBA1 co-staining decreased, whereas TSPO/GFAP (glial fibrillary acidic protein) co-staining increased over time (*p* < 0.001). Conclusion: We identify the inoculation-driven immune response to be a relevant contributor to the PET signal and add a new aspect to consider for planning PET imaging studies in orthotopic glioblastoma models.

## 1. Introduction

Glioblastomas are the most common primary malignant tumors of the central nervous system in adults and still have a devastating prognosis due to their invasive growth and recalcitrance [1,2]. Current research focuses on the characterization of the tumor microenvironment (TME) in glioblastoma using molecular imaging techniques [3,4]. Positron emission tomography (PET) imaging and a variety of innovative PET tracers targeting different functional regions within the tumor have gained increasing importance in the last two decades and help to decipher the heterogeneity of glioblastoma and its complex interplay with the TME [5,6,7,8]. In this context, orthotopic in vivo models of glioblastoma generated via the stereotactic inoculation of tumor cells into the brain of mice/rats are a valuable tool to validate non-invasive PET imaging findings in comparison to histological information, and consequently to help understand the underlying pathophysiological bases of imaging results [9,10,11].

Tumor-associated macrophages (TAMs) and microglia are the main cell entities of the TME for the imaging of glioma-associated inflammation, as both cell types show high upregulation in expression of the translocator protein (18 kDa) (TSPO) upon activation [12,13,14]. Hence, a wide range of TSPO PET tracers has been developed, initially mainly subjected to neuroinflammatory diseases [15]. For instance, the 3^rd^ generation TSPO radioligand [^18^F]GE-180 has shown promising results in preclinical studies performed on rodents and in human glioma PET studies [16,17,18,19]. Another imaging target in glioma is a subunit of the system-L amino acid transporter (LAT1) which acts as a general marker for tumor cells [20]. Currently, its radioligand *O*-(2-[^18^F]fluoroethyl)-L-tyrosine ([^18^F]FET) is routinely used for the diagnosis and disease monitoring of glioma in patients [21], as well as in preclinical research on glioblastoma in vivo models [22].

Although increasingly explored in glioblastoma, findings related to TSPO are complicated by the coincidence of its expression in both TAMs and tumor cells [10,23,24,25]. On the one hand, TSPO is known to be directly involved in glioblastoma hallmark features such as proliferation, migration and invasion [23]. On the other hand, TSPO is recognized as an imaging target for glioma-associated inflammation and reactive astrogliosis [26,27]. The coexistence and assumed crosstalk of these diverging roles of TSPO in glioblastoma are currently of high interest in order to better understand and enhance the interpretability of TSPO PET imaging results in glioblastoma.

The stereotactic inoculation process in orthotopic glioma mouse models is highly invasive and therefore represents a relevant inflammatory driver, which thus might interfere with the interpretability of TSPO PET data obtained in these models. To address this issue, we conducted a longitudinal dual tracer TSPO and amino acid PET study using the TSPO PET tracer [^18^F]GE-180 [28] in direct comparison to [^18^F]FET at days 7/8 and 14/15 after the orthotopic inoculation of GL261 murine glioblastoma cells (n = 24) or sham inoculation (n = 6) into the brains of fully immunocompetent C57BL/6 mice. In addition, sham-operated mice (n = 24) underwent [^18^F]GE-180 PET imaging at days 7, 14, 21, 28 and 35 to monitor potential reactive alterations that were solely related to the invasive inoculation process. In vivo imaging data were correlated with data obtained by autoradiography (ARG) and immunohistochemistry (IHC) to gain a detailed insight on the nature of cell populations that account for the positive TSPO PET signal of orthotopically implanted glioblastomas. Furthermore, a human TSPO PET scan of a glioma patient after stereotactic biopsy is presented in a translational perspective.

## 2. Materials and Methods

### 2.1. Study Design

In the first part of this work, we longitudinally compared [^18^F]GE-180 and [^18^F]FET uptake in immunocompetent C57BL/6 mice which were orthotopically implanted with GL261 murine glioblastoma cells. A total of n = 30 mice (n = 24 tumor mice/n = 6 sham mice), all female and 10 weeks old, underwent dual imaging with [^18^F]GE-180 PET (day 7 and 14 after implantation) and [^18^F]FET PET (day 8 and 15). To analyze regional radioligand distribution directly on the brain tissue at high resolution, intracardial perfusion, brain extraction and slicing for ex vivo [^18^F]FET ARG were performed immediately after the PET scan on day 8 (n = 6 tumor / n = 3 sham) or day 15 (n = 18 tumor / n = 3 sham). Additionally, in vitro [^18^F]GE-180 ARG and haematoxylin and eosin (H&E) staining were performed using directly adjacent slices. The detailed study design is shown in Supplementary Figure S1A.

In the second part of this work, we exclusively used sham-inoculated mice with the aim to monitor changes in TSPO-targeted imaging which were exclusively related to the inoculation process. Here, a total of n = 20 C57/BL6 mice were imaged on day 7, 14, 21, 28 and 35 after inoculation. For imaging analysis and tissue-based analyses, the unaffected hemisphere contralateral to the surgical site served as control. At each time point, n = 4 mice received a [^18^F]GE-180 PET followed by intracardial perfusion and brain extraction. Of those, n = 3 brains were sliced and received ex vivo [^18^F]GE-180 ARG shortly after the PET-scan. In vitro [^18^F]GE-180 ARG and IHC were additionally performed on these slices. N = 1 brain per time point was conserved for the enhanced quality of slices and IHC was performed. In a subsequent run, slices at day 50 and 90 after a sham inoculation of n = 5 mice (RAG2KO, 24 weeks old, female) received in vitro [^18^F]GE-180 ARG and IHC. A detailed illustration of the study design is provided in Supplementary Figure S1B.

### 2.2. Animal Model and Sham Injection/Tumor Inoculation

All animal experiments were performed in accordance with the FELASA (Federation of European Laboratory Animal Science Associations) guidelines, and approved by the local regulatory authority, Regierung von Oberbayern. Mice were kept under standardized laboratory conditions (12 h day/night cycle, 22 °C, 65% rH) with feed and water ad libitum*,* and inspected on a daily basis. The culturing of GL261 murine glioblastoma cells was performed as described previously [29]. Sham and orthotopic tumor inoculation into the right striatum were performed as previously described with slight modifications, the protocol is specified in the Supplementary Methods [29].

### 2.3. Positron Emission Tomography (PET)

The radiosynthesis of [^18^F]GE-180 and [^18^F]FET PET was performed as previously described [30,31]. Effectively 12.6 ± 2.1 Megabecquerel (MBq) of [^18^F]GE-180 or 13.8 ± 1.6 MBq of [^18^F]FET, dissolved in 150 µL saline, were injected into the tail vein and static PET scans were performed. Further details on the scan protocols are given in the Supplementary Methods.

Image analyses were performed using PMOD (PMOD Technologies Limited, Zurich, Switzerland). Sham mice [^18^F]GE-180 PETs of both parts of this work were pooled (n = 2 at day 7 and n = 1 at day 14 had to be excluded due to paravenous tracer injection). For the same reason, n = 1 tumor mouse [^18^F]GE-180 PET was excluded at day 7. For tracer uptake quantification in the tumor, two symmetrical volumes-of-interest (VOIs) of 0.05 cm^3^ each were defined for all [^18^F]GE-180 PETs: the first VOI_RH_ was placed in the anterior right hemisphere to enclose the tumor and the second VOI_LH_ was placed in the contralateral left anterior hemisphere to capture the unaffected cerebral background. For uptake quantification at the injection site in sham mice, a twofold approach was chosen. VOI_RH_top_ was defined at the superior brain edge to cover tracer spill-in from the meninges/skull and VOI_RH_bottom_ was set for uptake in the remaining right hemisphere at the inoculation channel (IC). VOI_LH_ in the left hemisphere was used as a measurement for unaffected background. Mean standardized uptake values (SUV_mean_), i.e., the mean regional radioactivity concentration corrected for injected dose and body weight, as well as their ratios with the background (target-to-background ratios, TBR_mean_) were calculated. The strictly symmetrical approach was chosen to factor out tracer uptake in areas characterized by intrinsically increased physiological uptake like the ventricular system. All scans were reviewed for potential spill-in of tracer uptake by surrounding structures such as the skull base. In none of the cases, tumor or IC tracer uptake significantly exceeded the right hemisphere.

### 2.4. Autoradiography (ARG), Haematoxylin and Eosin (H&E) Staining, and Immunohistochemistry (IHC)

A detailed protocol for intracardial perfusion and tissue preparation is given in the Supplementary Methods. For ex vivo ARG, brains were carefully removed from the skull and placed into a base mold embedded in cryo-matrix immediately after intracardial perfusion. After shock freezing for 10 min in dry ice, brains were fixed in a Leica CM 1510-1 Cryostat (Leica Microsystems, Nussloch, Germany) at −20 °C. Mouse brains of the dual tracer run with [^18^F]GE-180 and [^18^F]FET were cut into 16 µm transversal slices (n = 12 GL261 mice at day 15), or into coronal 8 µm slices to depict the inoculation area in different planes (n = 6 GL261 and n = 3 sham mice at day 8, n = 6 GL261 and n = 2 sham mice at day 15). The latter slices, in some cases, had limited tissue quality due to their relatively thin sections, resulting in tearing and rolling cryo-artefacts. To avoid such artefacts, the brains of the sham mice run until day 35 (n = 20) were cut into 25 µm coronal cryosections, and every 5^th^ and 6^th^ section was fixed onto a glass slide. Within 150 min after the PET scan with either [^18^F]GE-180 or [^18^F]FET, the slides were covered with a phosphor imaging plate (Fujifilm, Tokyo, Japan; BAS cassette2 2025) for a minimum of 12 h to generate ex vivo ARGs.

For in vitro ARG, either the same or adjacent slides as for ex vivo ARG were used, protocol details are given in the Supplementary Methods.

The obtained data were analyzed using AIDA image analyzing software (version 4.50; Elysia-raytest GmbH, Straubenhardt, Germany). The regions-of-interest (ROIs) of the IC were created via semi-automated hot-seed function, and in the case of adjacent physiological uptake they were manually adjusted using the polygon tool. For the volumetric approximation of the IC in sham mice, the Cavalieri method was applied [32]. Volumetric extrapolation was not performed in tumor mice that were cut in different planes. To estimate the [^18^F]GE-180 tracer uptake intensity at the IC, [^18^F]GE-180 uptake at the affected right hemisphere was divided by the uptake at the contralateral left hemisphere for slices displaying the IC (target-to-background ratios, TBR_mean_). H&E stainings were performed on similar slices as for in vitro ARG using a standard protocol [33].

For IHC, antibodies against TSPO, glial fibrillary acidic protein (GFAP), transmembrane protein 119 (TMEM119), ionized calcium-binding adapter molecule 1 (IBA1) and LAT1 were used. A detailed protocol of fluorescent IHC and confocal microscopy is given in the Supplementary Methods. The area-wise quantification of co-localized markers was chosen due to robustness against the different cell densities and sizes of the selected areas of interest. The regions of uptake in correlating ARG were used as landmarks for the extent of analyzed areas in IHC. Both slice-conserving methods showed similar results and therefore, data points obtained at each time point were pooled.

### 2.5. Statistical Analysis

Statistical analyses were performed with Graph Pad Prism (version 9.2.0 for Windows, GraphPad Software, San Diego, CA, USA). One-way analysis of variance (ANOVA) and Tukey’s multiple comparison test were used to compare the groups on days 7, 14, 21, 28, and 35 after sham inoculation. Student’s *t*-test was used for two-group comparisons. *p* < 0.05 was considered to be significant for a rejection of the null hypothesis.

## 3. Results

### 3.1. Tumor Mice

**Figure 1:**
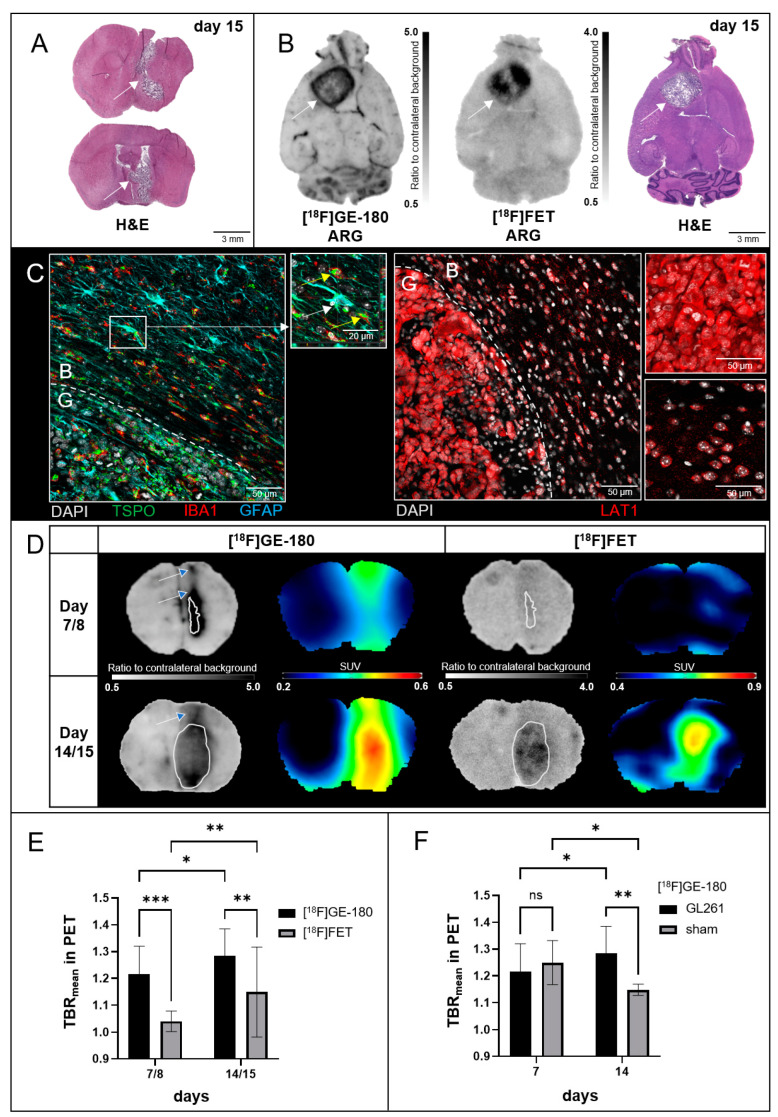
Comparison of [^18^F]GE-180 and [^18^F]FET uptake in GL261-implanted mice. (**A**) Coronal H&E slices show examples for alterations in tumor growth as the result of the inoculation process. Top: tumor cells expand along the inoculation channel. Bottom: tumor cells infiltrate the ventricular system and reach the skull base. White arrows point at the tumor. (**B**) Uptake at the tumor edge (in vitro [^18^F]GE-180 ARG, left, tumor marked by white arrow) versus uptake at the tumor center (ex vivo [^18^F]FET ARG, middle) at day 15 after tumor inoculation. Consecutive transversal slices, and a correlating HE slice are shown. (**C**) Immunohistochemistry showing co-staining of TSPO, IBA1 and GFAP (left) in a transversal slice of a GL261-inoculated C57/BL6J mouse at day 19 after tumor inoculation. B = peritumoral background, G = area containing glioma cells. High numbers of TSPO-labelled astrocytes (white arrow) and neuroinflammatory cells (yellow arrow) behind the glioma cell border (long dotted line) can be seen. Top left shows a z-stack caption of a reactive astrocyte. LAT1 staining (right) shows high binding at the tumor and low binding behind the glioma cell border (dotted line). Top right shows staining at the tumor center, bottom right at contralateral control. (**D**) Comparisons of [^18^F]GE-180 ARG with PET (left) and of [^18^F]FET ARG and PET (right) at day 7/8 (upper row) and day 14/15 (lower row) after inoculation in coronal view. [^18^F]GE-180 clearly shows additional uptake at the IC (arrows with blue arrowhead). White delineations mark the border of glioma cells at correlating HE staining. (**E**) SUV_mean_ of inoculated hemispheres divided by SUV_mean_ of contralateral unaffected hemispheres (=TBR_mean_) for [^18^F]GE-180 and [^18^F]FET PETs at day-7/8 (n = 23) and 14/15 (n = 17) after tumor inoculation. Student’s *t*-test: *, *p* < 0.05; **, *p* < 0.01; ***, *p* < 0.001. (**F**) SUV_mean_ of hemispheres with inoculated tumors/sham inoculated, divided by SUV_mean_ of contralateral unaffected hemispheres (=TBR_mean_) for [^18^F]GE-180 PETs for n = 23, n = 5, n = 17 and n = 6 from left to right. Student’s *t*-test: *, *p* < 0.05; **, *p* < 0.01, ns = not significant. Values are presented as mean ± standard deviation (SD).

To verify the presence and histological extent of tumors, H&E staining was performed (see Supplementary Figure S1 for the time points of brain extraction). All inoculated mice exhibited tumors. In n = 6/8 cases in coronal view, scattered glioma cells along the IC were observed at day 15 (see example for vertical growth along the IC in Figure 1A). An infiltration of glioma cells into the ventricular system was common (n = 13/18 at day 15).

Ex vivo [^18^F]FET and in vitro [^18^F]GE-180 ARG were used to assess regional radioligand distribution at high resolution and revealed different tracer uptake patterns in all mice at day 15 (see ARGs and corresponding H&E staining in Figure 1B). Whereas [^18^F]GE-180 regularly showed enhanced uptake along the tumor margin, [^18^F]FET uptake was more homogenous predominantly at areas of high cell density of the tumor core with some cutouts in areas of low cell density in the very central parts. The uptake extent of [^18^F]GE-180 partly surpassed [^18^F]FET and reached beyond the HE-positive dense tumor core. IHC analyses revealed, in concordance to the ARG findings, TSPO-labelled astrocytes and neuroinflammatory cells beyond the glioma margin while LAT1 was only highly expressed within the tumor (see Figure 1C).

Alterations in tumoral uptake patterns through the IC become visible when analyzing [^18^F]FET and [^18^F]GE-180 in coronal view. Figure 1D gives an example that at day 7/8, [^18^F]GE-180 PET/ARG (left) capture both tumor growth and alterations likely related to the inoculation process, while [^18^F]FET PET/ARG (right) display no noteworthy uptake at the inoculation site. At day 7, [^18^F]GE-180 PET showed, in contrast to ARG, prominent uptake at the superior brain edge. At day 15, the tumor occupies a substantial area when compared to day 8 and shows enhanced uptake in both, [^18^F]GE-180 and [^18^F]FET PET and ARG. Arrows with blue arrowheads mark the areas of exclusive [^18^F]GE-180 uptake beyond histological tumor border, likely caused by the inoculation process.

Figure 1E shows that the [^18^F]GE-180 uptake intensity at the inoculation hemisphere normalized to background exceeds corresponding [^18^F]FET uptake in PET at day 7 (TBR_mean_ 1.22 ± 0.10 vs. 1.04 ± 0.04, *p* < 0.001) and at day 14 (TBR_mean_ 1.28 ± 0.10 vs. 1.15 ± 0.04, *p* = 0.008).

Figure 1F displays TBR_mean_ in [^18^F]GE-180 PET. At day 7, uptake intensity did not differ significantly between tumor and sham mice (1.22 ± 0.10 vs. 1.24 ± 0.07, *p* = 0.637). However, contrary to sham mice, tumoral uptake increased over time (1.22 ± 0.10 vs. 1.28 ± 0.10, *p* = 0.047) resulting in significantly higher uptake in GL261-tumors when compared to sham mice at day 14 (1.28 ± 0.10 vs. 1.15 ± 0.02, *p* = 0.008).

### 3.2. Sham Mice

#### 3.2.1. PET

To verify the assumed inoculation-related impact on TSPO radioligand uptake found in the investigation of tumor mice, in a second step only sham-operated mice without tumor were used to monitor potential reactive alterations solely related to the invasive inoculation process.

**Figure 2:**
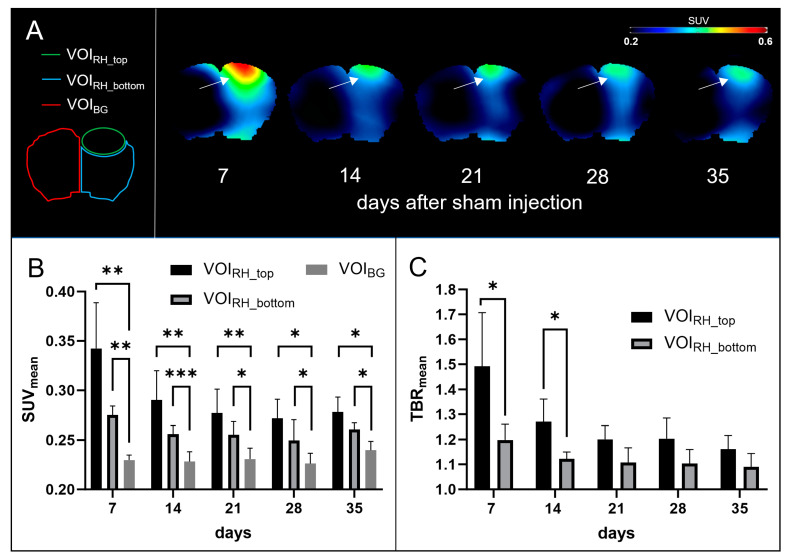
[^18^F]GE-180 PETs in sham mice. (**A**) On the left, VOI_RH_top_ (green), VOI_RH_bottom_ (blue) and VOI_LH_ (red) are projected on a healthy mouse brain in coronal view. On the right, representative PETs of sham-operated mice are shown. White arrows point at the highest signal at the superior brain edge, probably related to meningeal uptake. (**B**) SUV_mean_ of day 7 (n = 5), day 14 (n = 6), day 21 (n = 4), day 28 (n = 4) and day 35 (n = 4). Student’s *t*-test: *, *p* < 0.05; **, *p* < 0.01; ***, *p* < 0.001. (**C**) TBR_mean_ for VOI_RH_top_ and VOI_RH_bottom_. One-way ANOVA *p* = 0.004 for VOI_RH_top_, and *p* = 0.04 for VOI_RH_bottom_. Student’s *t*-test: *, *p* < 0.05. Values are presented as mean ± SD.

Here, PET-imaging revealed increased [^18^F]GE-180 uptake at the injection site, which in all sham mice was rather diffuse and without a sharp demarcation of the IC. Figure 2A shows representative PETs in coronal view. It was noteworthy that the markedly elevated uptake at the superior brain edge, previously also found in tumor mice, is located slightly below the physiological skull uptake of [^18^F]FET PET. Figure 2B clarifies that uptake at the right hemisphere (VOI_RH_top_ and VOI_RH_bottom_) was significantly higher than background uptake at days 7, 14, 21, 28, and 35 (all *p* < 0.05). At early time points, uptake at the top near the skull was significantly higher than bottom uptake. Figure 2C shows corresponding SUV_mean_ ratios, which both significantly decrease over time (one-way ANOVA, *p* = 0.004 for VOI_RH_top_, and *p* = 0.04 for VOI_RH_bottom_).

#### 3.2.2. ARG

**Figure 3:**
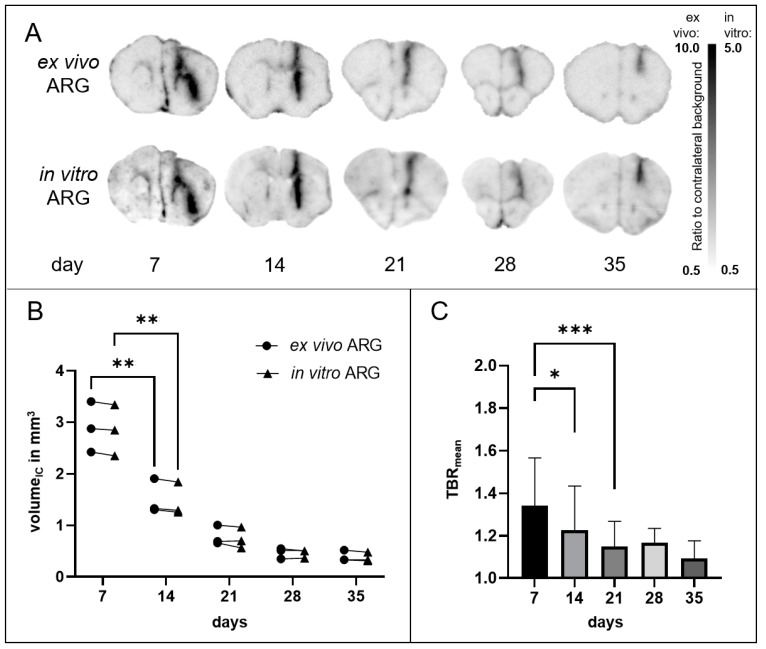
[^18^F]GE-180 autoradiographies (ARGs) in sham mice. (**A**) Representative ARGs at different time points after sham injection in coronal view. Ex vivo and in vitro ARGs show nearly congruent signals with easily delineable IC. (**B**) Volumes in mm^3^ of ex vivo (circles) and in vitro (triangles) ARGs. Pairwise data of each mouse are connected by lines. One-way ANOVA for ex vivo ARG *p* < 0.001 and in vitro ARG < 0.001. Tukey Test: **, *p* < 0.01. (**C**) Tracer uptake intensity in right hemispheres divided by intensity in left hemispheres (=TBR_mean_) for slices displaying the IC at day 7 (n = 38 slices), 14 (n = 29), 21 (n = 23), 28 (n = 19), and 35 (n = 22) after sham injection. One-way ANOVA *p* < 0.001. Tukey Test: *, *p* < 0.05; ***, *p* < 0.001. Values are presented as mean ± SD.

Ex vivo [^18^F]GE-180 ARG was used to verify in vivo PET imaging findings at high resolution on brain tissue. In all sham mice, ARG showed increased uptake at the injection site. Regional tracer distribution in ex vivo ARG was nearly congruently duplicated in in vitro [^18^F]GE-180 ARG (e.g., see Figure 3A). The uptake visually decreased over time, especially in deeper brain areas and less on the brain surface, but could still be detected in 8.7 ± 2.5 slice levels per mouse with an interval of 150 µm each. The calculated approximate volume of the IC did not differ significantly in ex vivo and in vitro ARG (Figure 3B). Furthermore, it decreased significantly within the five weeks after sham injection (one-way ANOVA *p* < 0.001 for ex vivo ARG, *p* < 0.001 for in vitro ARG) (Figure 3B). The uptake intensity at the IC also significantly decreased over time with highest loss at early time points between days 7 and 14 (one-way ANOVA *p* < 0.001) (Figure 3C). In vitro ARG at day 50 and 90 after sham injection still revealed a low [^18^F]GE-180 uptake at the IC (see Supplementary Figure S2).

#### 3.2.3. Immunohistochemistry (IHC)

**Figure 4:**
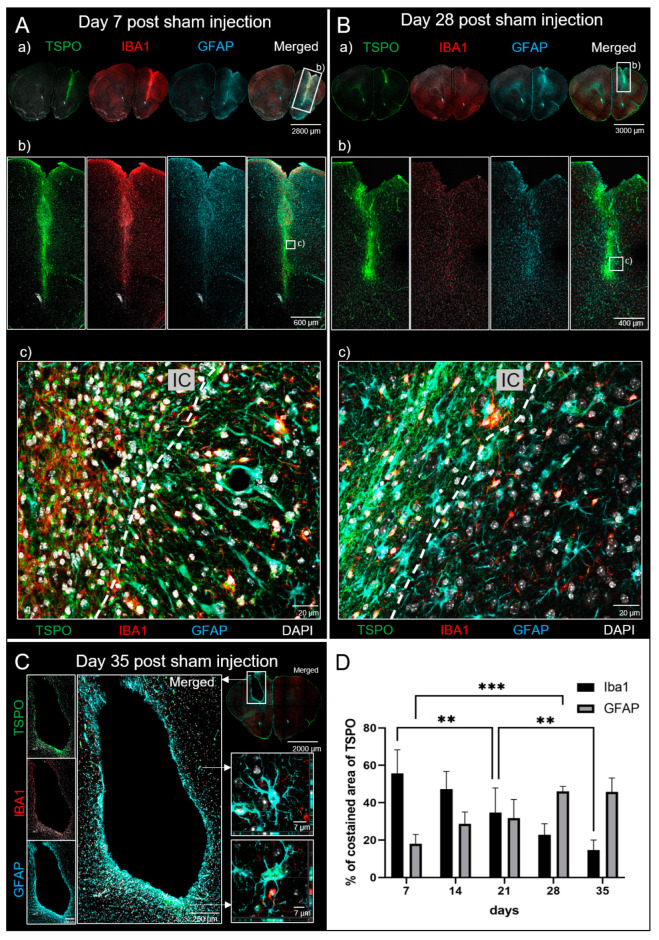
TSPO, IBA1 and GFAP co-staining in sham mice. (**A**) Representative coronal IHC pictures at day 7 after sham injection showing a (a) brain overview, (b) zoom-in at IC and (c) detailed view of the IC edge at cellular level. All shown channels are merged with DAPI. Dotted lines mark the IC border. (**B**) Pictures at day 28 after sham injection in the same arrangement as in (**A**). IBA1 signal at the IC is clearly decreased compared to day 7 while TSPO and GFAP signals remain strong. (**C**) Rim-like structure of TSPO-positive astrocytes surrounding a cavity in the brain at the previous injection site 35 days after sham injection. The middle right picture shows the z-stack of a TSPO-negative astrocyte, while the bottom right picture shows the z-stack of a TSPO-labelled astrocyte. (**D**) Proportion of co-stained areas of IBA1 and GFAP with overall areas of TSPO expression at the IC at day 7 (n = 3), 14 (n = 3), 21 (n = 3), 28 (n = 3), and 35 (n = 2) with 2–3 analyzed slices per mouse. One-way ANOVA for IBA1 *p* < 0.001 and GFAP *p* < 0.001. Tukey Test: **, *p* < 0.01; ***, *p* < 0.001. Values are presented as mean ± SD.

TSPO, IBA1 and GFAP co-staining showed dynamic binding patterns during the first five weeks. Figure 4A,B representatively compares day 7 and 28. The TSPO signal along the IC is highly elevated in comparison to the contralateral control. Neuroinflammatory cells (IBA1/TSPO co-labelled cells) and reactive astrocytes (GFAP/TSPO co-labelled cells) were identified as the main source of TSPO signal. The prominent TSPO expression in IBA1-positive (IBA1+) cells was condensed and perinuclear. In reactive astrocytes, TSPO was more diffusely distributed, also reaching to their branches. Both cell types showed high density, especially at the injection site, and were commonly found in juxtaposition. Interestingly, a shift in the proportion over time of both co-labelled cell types was detected. Neuroinflammatory cells contributed to the majority of TSPO signal during the first two weeks and then they gradually diminish. On the contrary, at later time points astrocytes were the main source of TSPO signal. They also increasingly formed well-organized formations, for instance rim-like structures surrounding an empty cavity at the sham injection site at day 35 (see Figure 4C). Figure 4D displays the quantitative shift in the contribution to overall TSPO signal from neuroinflammatory cells to astrocytes.

The microglia marker TMEM119 displayed very weak binding to cells at the IC in the first two weeks (see Supplementary Figure S3A). In contrast, at this area often group-wise arranged IBA1+ cells were found and identified as macrophages. At later time points, the total amount of IBA1-labelled cells at the IC was decreased while increasingly microglia could be identified (see Supplementary Figure S3B). At the contralateral background nearly all IBA1+ cells co-stain with TMEM119 (see Supplementary Figure S3C). Quantification of co-localized areas confirmed the observed longitudinal dynamics of inflammation cells at the IC (see Supplementary Figure S3D).

Supplementary Figure S4 illustrates IHC at the IC and corresponding [^18^F]GE-180 ARG. Supplementary Figure S5 correlates the area of TSPO staining and co-localized areas with IBA1 and GFAP staining to uptake intensity at the IC in in vitro [^18^F]GE-180 autoradiography.

Supplementary Figure S2 shows IHC on brain slices of day 50 and 90 after sham injection. At both time points, high numbers of well-organized reactive astrocytes that co-stained with TSPO were found at the IC. Although the RAG2KO model is known to lack regular B and T cell maturation [34], at day 50, IBA1 is slightly elevated when compared with healthy brain and there are still macrophages located at the IC.

### 3.3. The TSPO PET-Scan of a Glioma Patient after Stereotactic Biopsy

**Figure 5:**
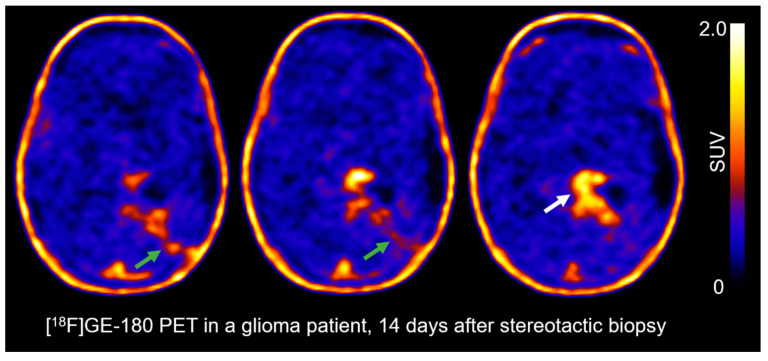
Increased uptake along the biopsy trajectory in a human [^18^F]GE-180 PET. Transversal view of three adjacent sections side-by-side of the same PET scan with an interval of 4 mm each. TSPO PET was performed 14 days after serial stereotactic biopsy. A glioma recurrence was confirmed in the left parasagittal area with focally increased uptake (white arrow). Additionally, an increased radioligand uptake is noted along the biopsy trajectory (green arrows).

Following the analysis of the preclinical data, similar PET findings were noted in a human glioma case. Figure 5 shows the TSPO PET scan of a glioma patient 14 days after stereotactic biopsy. A total of 170 MBq [^18^F]GE-180 were injected intravenously and emission data were acquired 60–80 min p.i. The PET images show a focally increased TSPO radioligand uptake in the left parasagittal area (white arrow) and a glioma recurrence was confirmed in neuropathological analyses. Beyond the latter, however, an increased TSPO radioligand uptake was also noted along the biopsy trajectory (green arrows).

## 4. Discussion

We conducted a longitudinal dual tracer TSPO and amino acid PET study with [^18^F]GE-180 and [^18^F]FET in direct comparison to tissue-based analyses in glioblastoma-bearing and sham-operated mice to assess the impact of the inoculation process on TSPO PET in the frame of experimental orthotopic glioblastoma. We found that the invasive inoculation process alters healthy brain tissue and consequently influences TSPO radioligand uptake, however this inherent feature of stereotactic inoculation can barely be found in the literature yet. Therefore, we want to raise awareness that the impact of the inoculation process should be paid attention to when analyzing the TME in orthotopically implanted syngeneic mouse models of glioblastoma, at least at early time points after surgery. Furthermore, we provide information about the longitudinal changes of TSPO PET findings and of TSPO-positive cell populations after penetrating traumatic brain injury (TBI) and underscore the validity of the TSPO radioligand [^18^F]GE-180 in a preclinical setting.

In clinical glioma studies, TSPO radioligand uptake in PET regularly exceeded [^18^F]FET uptake [18,26]. This matches with the discordant uptake patterns of [^18^F]FET and [^18^F]GE-180 in the present study. While [^18^F]FET was restricted to the tumor, [^18^F]GE-180 uptake was also markedly increased at the tumor infiltration zone and further uptake along the IC was detected, which conflated with the tumoral uptake. Even though tumors repeatedly showed a tendency to grow vertically along the IC, which thus does not correspond to a natural growth situation, increased uptake well above the superior tumor margin was noted in all cases, here pointing towards a non-tumoral origin of increased TSPO expression. [^18^F]GE-180 PET and ARG in sham-operated mice without tumor eventually substantiated such uptake to be related to the inoculation process. Here, a markedly increased focal extracerebral [^18^F]GE-180 uptake—probably related to the meninges as not present on [^18^F]GE-180 ARG and located below skull uptake in correlation to FET imaging—and a less increased uptake along the intracerebral IC were noted. Although the increased TSPO radioligand uptake along the IC was noted until day 35 after sham injection, it significantly decreased over time, especially from day 7 to day 14. Considering the overall survival time of untreated GL261-bearing mice of about 21 days until neurological symptoms appear, it is especially the early time points that are regularly of interest for PET imaging and are intended to reflect the TME in the condition of tumor growth initiation [25,35]. However, at day 7, [^18^F]GE-180 uptake in this orthotopic model was mainly driven by the inoculation process. In contrast, at day 14, [^18^F]GE-180 PET and ARG were already dominated by tumor-associated uptake, and the results in sham mice overall indicated that the inoculation process had less impact on TSPO radioligand uptake at later time points. Hence, the time window to monitor the glioma-inherent TSPO signal might be rather short in the GL261 model. This might be different for other orthotopic glioma models. In the SB-28 glioblastoma model, which also uses immunocompetent C57BL/6 mice, the magnitude of the tumoral TSPO radioligand uptake exceeds by far the inoculation-induced uptake at early time points, as recently shown [36], warranting comparative analyses on the intrinsic TSPO protein expression levels in the GL261 and SB-28 cell lines and on the potentially distinct degree of TAM infiltration in both models. Also, the inoculation of a reduced amount of tumor cells might enable longer survival and therefore increase late time points for in vivo imaging. The combinatorial settings of TSPO-wildtype/knock-out host mice transplanted with TSPO-wildtype/knock-out tumor cells could help to better understand the contribution to the TSPO signal. By comparing the TSPO radioligand uptake of GL261 in TSPO+/+ and TSPO−/− mice, Banati et al. found that the TSPO PET signal extended beyond the tumor in TSPO+/+ mice as opposed to TSPO−/− mice, thus heavily supporting the assumption of a relevant inflammatory component to the TSPO signal [37]. Still, a complex bidirectional crosstalk between tumor cells and TAMs is described, and it remains unclear how far neuroinflammatory cells along the IC differ from TAMs and interact with glioma cells [38,39]. The inflammatory milieu caused by the inoculation process may alter the tumor pathology compared to endogenous tumors in properties like growth and signaling behavior [40,41]. Also, the pronounced TSPO tracer uptake at the tumor edge does not necessarily originate from tumor-induced inflammation but might represent inflammatory cells initially recruited and activated during the inoculation process which were merely pushed and concentrated at the tumor edge following tumor growth. The needle size used for the implantation of tumor cells also has to be considered in this context: glass pipettes with a fraction of the diameter of conventional needles are available in the field of patch-clamp technology and were adopted as an injection tool in the field of 2-photon microscopy [42]. Meanwhile, 3D-printing tools allow for lower-cost production of similar glass pipettes, suitable for atraumatic cell injection [43]. These could also be used for the orthotopic inoculation of glioblastoma cells and hence reduce needle-related limitations including potential backflow along the needle track. Here, metastatically induced tumor models or genetic models which develop glioblastomas spontaneously might better replicate natural tumor growth conditions [44]. Furthermore, the inoculation of non-tumoral cells as a sham control instead of saline might enable the assessment of changes in TSPO expression induced in the healthy brain by grafted cells in general, independently of neoplastic features and malignant growth.

While the time point of PET imaging influences the relative impact of the inoculation process on TSPO tracer uptake in the GL261 model, several other factors may complicate the in vivo imaging of tumoral TSPO expression. Firstly, factors influencing the host immunity are likely significant, including its genetic background or its sex and age. Zinnhardt et al. mentioned TSPO immunoreactivity along the IC on day 14 using female NMRI nu/nu mice [45]. The immunodeficient background of these mice may be the cause for the visually less pronounced IC compared to our work, although NMRI mice, such as athymic mice, have a rather low level of immunodeficiency when compared to B6RAG2 or NOD-scid mice, in which a variety of immune cells including substantial myeloid populations are absent or dysfunctional [46]. Additionally, specific features of microglia by exposure to different sex steroids and age-associated priming for microglia activation are described [47,48]. Secondly, factors that occur during the surgery like skull penetration, anti-inflammatory medication, needle thickness or daytime inoculation may alter the IC and impact TSPO expression [49]. We used metamizole, which is likely to have altered the immune response to the needle penetration [50]. The injection site directly at the cortex surface showed enhanced [^18^F]GE-180 uptake, especially in PET. Cai et al. described meningeal lymphatic vessels as a possible entry point of myeloid cells after acute brain injury [51]. Consequently, skull penetration should be performed with caution to minimize meningeal damage. Multi-omics approaches and whole mouse transparency recently helped unravel in more depth the pathophysiological interplay between skull alterations and TSPO uptake patterns mirroring the underlying neuroinflammation [52].

Tissue-based analyses in tumor-free sham-injected mice revealed a shift over time in the contribution to the overall TSPO signal from mainly microglia/macrophage activation at early time points to astrogliosis at late time points after penetrating TBI. We found IBA1+ cells in juxtaposition to reactive astrocytes at the IC, however they were found at varying amounts over time. Low TMEM119 binding during the first two weeks is indicative of a low proportion of homeostatic microglia and therefore compatible with a higher amount of disease-associated microglia [53]. Turtzo et al. described a peak of macrophage/microglial response at 5–7 days post-TBI and strongly decreasing inflammation-associated ribonucleic acid (RNA) expression within the first weeks in a rat TBI model [54]. In concordance, we observed a significantly decreasing proportion of IBA1 signal from overall TSPO signal within the first weeks. At day 90, there was visually no difference in IBA1 expression at the IC compared to the contralateral background. Mishra et al. found that, in their TBI model, the numbers of infiltrating macrophages outweighed resident microglia at the inflammation site [55]. However, this study did only monitor 72 h after insult. Our results identify macrophages as the dominant inflammatory cell type until day 35 post-TBI. A brief additional discussion on rat models and TBI can be found in the Supplementary Discussion.

In the context of the TME, a lot of studies focus on the TSPO expression primarily in neuroinflammatory cells [10,45], whilst the contribution of reactive astrocytes to the overall TSPO signal is often not examined or only attributed subordinated importance [26,56,57]. Nevertheless, astrocytes are an essential part of the natural TME, and a role of TSPO in reactive astrocytes has been known for more than fifteen years [58,59]. As shown in the present study, astrocytes can be a relevant additional source of TSPO expression contributing to the TSPO radioligand uptake at tumor edges. Intriguingly, more sophisticated, innovative methods of TSPO uptake quantification at cellular resolution will help further disentangle the contribution of distinct cellular entities to the overall TSPO signal and are underway [60,61].

There is a potential that stereotactic biopsies in humans may cause similar TSPO-related reactive effects as the inoculation process in rodents and should be paid attention to, as the application of TSPO PET in glioma patients is an area of continuously increasing interest [8]. Figure 5 shows increased uptake along the biopsy trajectory in a human TSPO PET. Its origin in this case remains unclear but, on a translational notion, a biopsy-induced immune response could be hypothesized as the cause, following the preclinical findings of this work. In the GL261 mouse model, we prove [^18^F]GE-180 to be an excellent marker for TSPO-expressing cells, as ex vivo and in vitro [^18^F]GE-180 ARG showed in all cases (n = 15) highly congruent uptake, and as [^18^F]GE-180 uptake correlated with TSPO-labelled areas in IHC. A PET-biopsy study in human glioma patients, correlating the areas of increased [^18^F]GE-180 uptake with tissue-based analyses, is currently in progress.

## 5. Conclusions

We identify the invasive inoculation process used for generating orthotopic glioblastoma in vivo models to be a relevant contributor to the TSPO radioligand uptake in the GL261 glioblastoma mouse model. Furthermore, we provide information about the longitudinal changes of TSPO PET findings and the underlying TSPO-positive cell populations after penetrating traumatic brain injury. Less traumatic models, e.g., using glass pipettes for the injection of tumor cells, may alleviate the injection-related concerns for PET imaging. This adds a new aspect to consider for planning PET imaging studies using the orthotopic GL261 glioblastoma model.

## Data Availability

The core of the data is gathered in the manuscript and the Supplementary Materials. Further data may be available from the corresponding author on reasonable request.

## Supplementary Materials

The following supporting information can be downloaded at: https://www.mdpi.com/article/10.3390/biomedicines12010188/s1, Supplementary Methods, Supplementary Discussion, Figure S1: Graphical illustrations of study designs; Figure S2: Immunohistochemistry and in vitro [^18^F]GE-180 autoradiography (ARG) on slices of day 50 (left) and day 90 (right) after sham injection; Figure S3: IBA1 and TMEM119 co-staining in sham mice; Figure S4: Correlation of [^18^F]GE-180 autoradiography (ARG) with TSPO, IBA1 and GFAP co-staining; Figure S5: Scatterplots of in vitro [^18^F]GE-180 autoradiography intensity in QL/Bq/mL and area of immunohistochemistry (co-)staining in ppi^2^ with regression line and 95% confidence interval. References [19,22,29,62,63,64,65,66,67,68,69,70] are cited in the supplementary materials.
