# Supplementary material for: The Traumatic Inoculation Process Affects TSPO Radioligand Uptake in Experimental Orthotopic Glioblastoma
Source: Biomedicines. 2024 Jan 15;12(1):188. doi: 10.3390/biomedicines12010188 (PMC10813339; doi:10.3390/biomedicines12010188)
Supplement: Supplementary file 1 [file biomedicines-12-00188-s001.zip › biomedicines-2796530-supplementary.pdf]

Impact of inoculation-driven immune response on PET imaging in experimental orthotopic glioblastoma

by

Lukas Gold [1*], Enio Barci [2*], et al., and Adrien Holzgreve [1]

Short running title: Impact of orthotopic inoculation on glioblastoma PET signal

Corresponding Author:
Dr. med. Adrien Holzgreve, MHBA
Department of Nuclear Medicine
LMU University Hospital, LMU Munich
Marchioninistr. 15, 81377 Munich, Germany
Phone: +49 89 4400 74665
E-Mail: Adrien.Holzgreve@med.lmu.de

Supplementary methods

Sham injection / tumor inoculation

After pre-medication and anesthesia with 200 µg/g metamizole, 100 µg/g ketamine, and 10 µg/g xylazine (all from WDT, Garbsen, Germany) mice' heads were mounted on a stereotactic frame in a flat-skull position. Bepanthen cream was used for eye protection and scalps were disinfected using 7.5 % povidone-iodine solution. Skulls were exposed by a longitudinal, sagittal skin incision and a hole was drilled 1.5 mm laterally and 1 mm anteriorly to the bregma using a 21 G microlance. Then, a 22 G Hamilton syringe was inserted perpendicularly into the skull to a depth of 4 mm starting from the dura surface before retracted by 1 mm to create a vacuity for the injection. 1 µl of either 0.9 % saline solution (sham mice) or 0.9 % saline solution containing 100,000/µl GL261 cells (tumor mice) were injected into the right striatum. After injecting half of the suspension, one minute resting time followed. The needle was then retracted with a pace of 1 mm/min while injecting the rest of the suspension. Finally, the skin was cleaned and sutured, and mice were monitored until regaining consciousness.

Positron emission tomography (PET)

Anesthesia was performed with 2 % isoflurane delivered via a mask at 3.5 L/min in oxygen. Up to four mice were placed simultaneously in the tomograph (Siemens Inveon DPET, Siemens, Erlangen, Germany). For [^{18}F]GE-180 PET, a transmission scan was performed 45-60 min post injection (p. i.), and an emission scan was recorded 60-90 min p. i.. For [^{18}F]FET PET, an emission scan was recorded 20-40 min p. i., and a transmission scan was performed 40-60 min p. i.. Image reconstruction procedure, scattering and attenuation correction were performed as previously described [1,2].

All PETs were automatically fused onto a suitable PET template using the RIGID matching tool in PMOD with default parameters, and the fusion were afterwards checked and corrected if necessary. All [^{18}F]FET PET scans were normalized to body masses and applied tracer activity, given in g/ml. All [^{18}F]GE-180 PET scans were scaled to myocardial tracer uptake as previously established [3].

Intracardial perfusion and tissue preparation

Sterilized 0.01 M phosphate-buffered saline (PBS), followed by 4% Paraformaldehyde (PFA) solution were used for intracardial perfusion of the animals. For IHC, brains were incubated in 4% PFA for 48 h at 4 °C, then dehydrated in 30% sucrose (w/v) in 0.01 M PBS until the sample sank to the bottom of the falcon tube. Finally, the brains were embedded in Cryomatrix® (ThermoFisher Scientific, cat.-nr. 6769006) and frozen in liquid nitrogen. 40 µm coronal sections were obtained by a sliding microtome (PFM AG, Cologne, Germany) and successively collected in 24-well plates that were filled with cryoprotectant fluid (glycerol, ethylene glycol, and 0.01 M PBS at ratio 1:1:2). The plates were stored at -20 °C protected from light.

In vitro autoradiography (ARG)

Tissue was covered with a solution of [^{18}F]GE-180 and 5% (w/v) TRIS-buffer (approximately 3 MBq / 50 ml). After incubation for 1h, the slides were washed per rinsing by ice cold 5% TRIS-

buffer for 20 seconds and directly afterward dipped into distilled water for 10 seconds. Then, slides were dried for about 45 minutes and placed under imaging plates for a minimum of 12h.

<u>Fluorescent immunohistochemistry and confocal microscopy</u>

The free-floating sections were washed three times, 5 min each, in PBS containing 0.1 % Tween® 20 (PBST) using a 12-well plate in a rotary shaker. The protein blocking process was conducted for 1 h at room temperature with 5 % normal donkey serum in PBS containing 0.3 % Triton X-100 (Sigma-Aldrich, cat.-nr. 9036-15). The sections were then incubated at 4 °C overnight with primary antibody solutions. The primary antibodies used in this study were: rabbit anti-PBR antibody 1:200 (Abcam, cat.-nr. ab109497), rabbit anti-TMEM119 1:200 (Abcam, cat.-nr. ab20964), chicken anti-GFAP 1:500 (Abcam, cat.-nr. A85307), goat anti-IBA1 1:200 (Abcam, cat.-nr. ab5076), and rabbit anit-LAT1 1:200 (Biozol, cat.-nr. LS-C415524). The next day, after repeated washing as described above, the sections were incubated with secondary antibody solutions for 2 h at room temperature. The secondary antibodies used in this study were: donkey anti-rabbit AF488 1:500 (ThermoFisher Scientific, cat.-nr. A-21206), donkey anti-goat AF594 1:500 (ThermoFisher Scientific, cat.-nr. A-11058), and donkey anti-chicken AF647 1:500 (ThermoFisher Scientific, cat.-nr. A78952). After washing the sections with PBST, sections were mounted on glass slides and air dried for 10 min. Nuclei were stained with DAPI (1:10,000) for 3 min at room temperature, followed by a short rinse in aqua destillata. 3 slices with an interval >240 µm per staining and mouse were stained for each time point.

The sections obtained with the cryostat were stored at -80 °C, and only consecutive slices of the ARG were stained according to the IHC protocol. However, incubation and washing steps were performed directly on the glass slides since the sections were already mounted.

Confocal microscopy was performed using a TCS SP8 microscope (Leica Microsystems GmbH, Wetzlar, Germany), and image z-stacks were processed with LAS Montage Imaging Software (Leica Microsystems GmbH, Wetzlar, Germany) and Image-J Software (NIH, Bethesda, MD, USA).

Supplementary discussion

Rat models and traumatic brain injury (TBI)

Besides their recognition as markers primarily of activated microglia/macrophages, TSPO-radioligands have been recognized as potential astrogliosis markers [4,5]. We observed well-organized structures of the astrocytes at the IC, especially at later time points. This is in concordance with previously described rim-like structures of astrocytes surrounding the core of infarction in a focal ischemia model in rats [6]. Wang at al. made similar observations of an increased TSPO tracer uptake in PET in a rat model of TBI [7]. In a rat model with focal cerebral ischemia, a peak of TSPO tracer uptake at day 11 has been described [8]. Also, both studies showed increased tracer uptake still several weeks after insult but did not identify the time point when it got back to the same level as control. In concordance, we found that *in vitro* ARG showed enhanced [^{18}F]GE-180 uptake 50 and even 90 days after TBI, here primarily related to astrogliosis as elucidated in the main manuscript. However, the comparability of those findings may be limited by the differing immune landscape of the models used and their distinct abilities to recruit immune cells [9,10]. Stereotactic inoculation is, besides in mice, also common in rat models [11,12]. Because the rat brain and consequently its tumors are significantly larger than in mice, the IC may relatively have less impact on the overall TSPO-radioligand uptake in orthotopic experimental glioma. Nevertheless, in rats too TBI should be considered as a potential mitigator of PET findings thought to display tumoral TSPO expression. Also in non-tumoral rat models, elevated TSPO tracer uptake could be observed in figures along the injection tract on PET, which however was not specifically mentioned or further elaborated on by the authors [5].

Supplement captions:

Supplementary Figure S1 | Graphical illustrations of study designs. (**A**) Longitudinal, dual [^{18}F]GE-180 vs. [^{18}F]FET imaging in the orthotopic GL261 mouse model. (**B**) Longitudinal monitoring of the IC in sham inoculated mice. [1]Orthotopic inoculation of the suspension into the right frontal lobe. [2]Brain immersion in 30% sucrose solution after fixation with 4% PFA to reduce artefacts from the shock freezing process.

Supplementary Figure S2 | Immunohistochemistry and *in vitro* [^{18}F]GE-180 autoradiography (ARG) on slices of day 50 (left) and day 90 (right) after sham injection. (**A**) Representative TSPO, IBA1 and GFAP co-staining at day 50 after sham injection in a) zoom-in at the IC and b) detailed view of the IC edge at cellular level. All shown channels are merged with DAPI. Dotted lines mark the IC border. (**B**) Representative TMEM119 and IBA1 co-staining at day 50 after sham injection. Yellow arrows point at microglia, blue arrows at macrophages. (**C**) and (**D**) Representative IHC at day 90 after sham injection in same arrangement as in (**A**) and (**B**). (**E**) and (**F**) Yellow arrows point at uptake at the IC in *in vitro* [^{18}F]GE-180 autoradiography. Slicing artefacts can be observed below the IC.

Supplementary Figure S3 | IBA1 and TMEM119 co-staining in sham mice. (**A**) Representative coronal IHC pictures at day 7 after sham injection in a) brain overview, b) zoom-in at IC, and c) detailed view of the IC edge at cellular level. All shown channels are merged with DAPI. Yellow arrows point at microglia. Blue arrows point at macrophages. Dotted lines mark the IC border. (**B**) Pictures of day 28 after sham injection in same arrangement as in (A). IBA1 signal at the IC is compared to day 7 showing clear decrease while more IBA1 and TMEM119 co-stained cells can be identified. (**C**) Contralateral background of the IC at day 7 post sham injection. Top right picture shows a z-stack of microglia. (**D**) Proportion of co-stained area of TMEM119 with overall area of IBA1 expression at the IC and contralateral background (BG) at day 7 (n=3), day 14 (n=3), day 21 (n=3), day 28 (n=2), and day 35 (n=2) with 2-3 analyzed slices per mouse. One-way ANOVA for proportion of TMEM119 at the IC $p<0.001$. Tukey Test: ***, $p<0.001$.

Supplementary Figure S4 | Correlation of [^{18}F]GE-180 autoradiography (ARG) with TSPO, IBA1 and GFAP co-staining. (**A**) Representative coronal IHC picture at day 21 after sham injection in detailed view of the IC edge at cellular level. At the IC, TSPO shows co-staining with IBA1 and GFAP. All shown channels are merged with DAPI. Dotted line marks the IC border. (**B**) Uptake of *ex vivo* and *in vitro* [^{18}F]GE-180 ARG on the consecutive slice reveal great correlation with TSPO labelled area in IHC.

Supplementary Figure S5 | Scatterplots of *in vitro* [^{18}F]GE-180 autoradiography intensity in QL/Bq/ml and area of immunohistochemistry (co-)staining in ppi^2 with regression line and 95% confidence interval. (**A**) Pearson´s r = 0.6819, p=0.0003; Spearman’s r = 0.7599, p<0.0001. (**B**) Pearson´s r = 0.8320, p<0.0001; Spearman’s r = 0.8394, p<0.0001. (**C**) Pearson´s r = 0.8488, p<0.0001; Spearman’s r =0.8547, p<0.0001.

Supplementary Figure S1

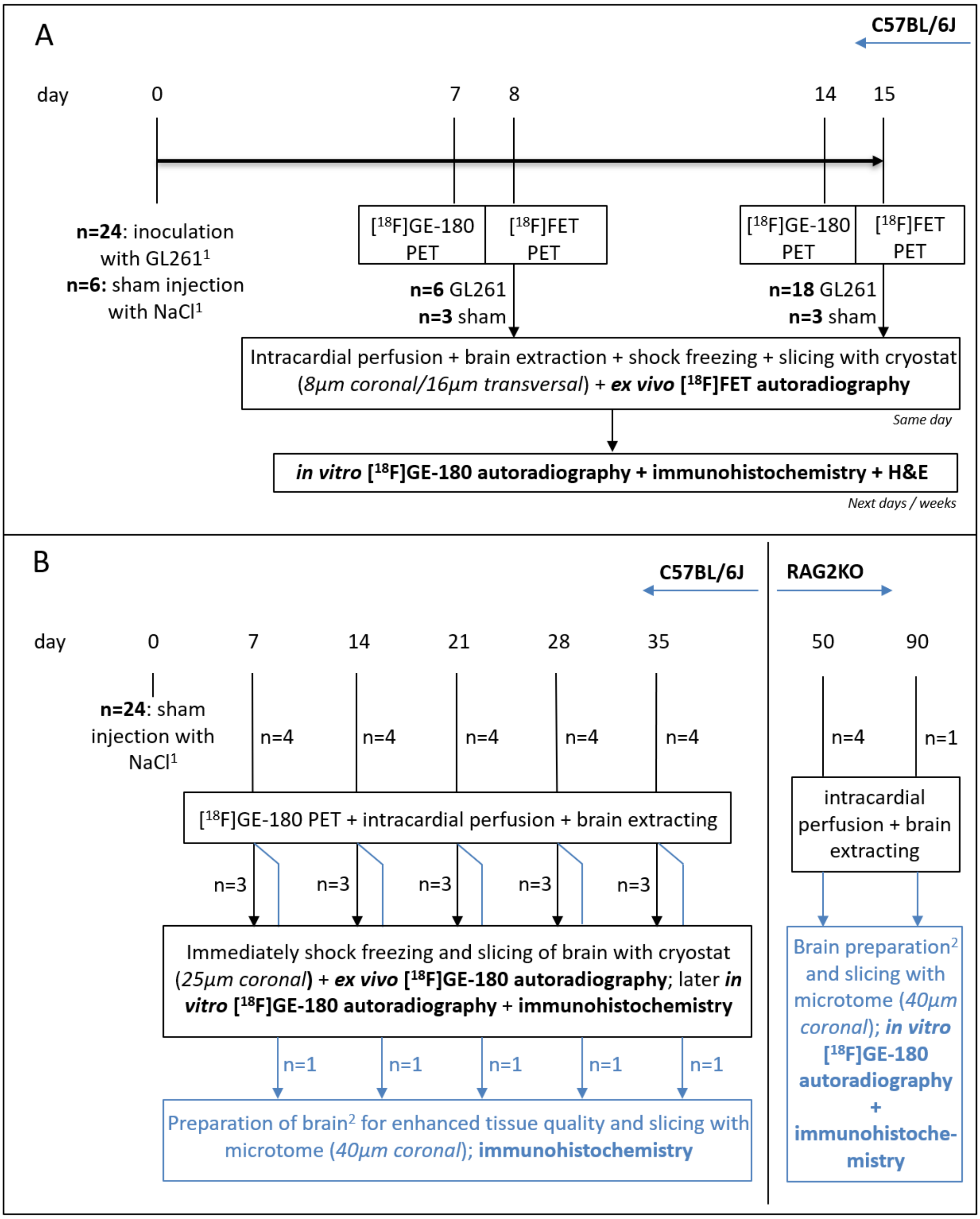

170 **Supplementary Figure S2**

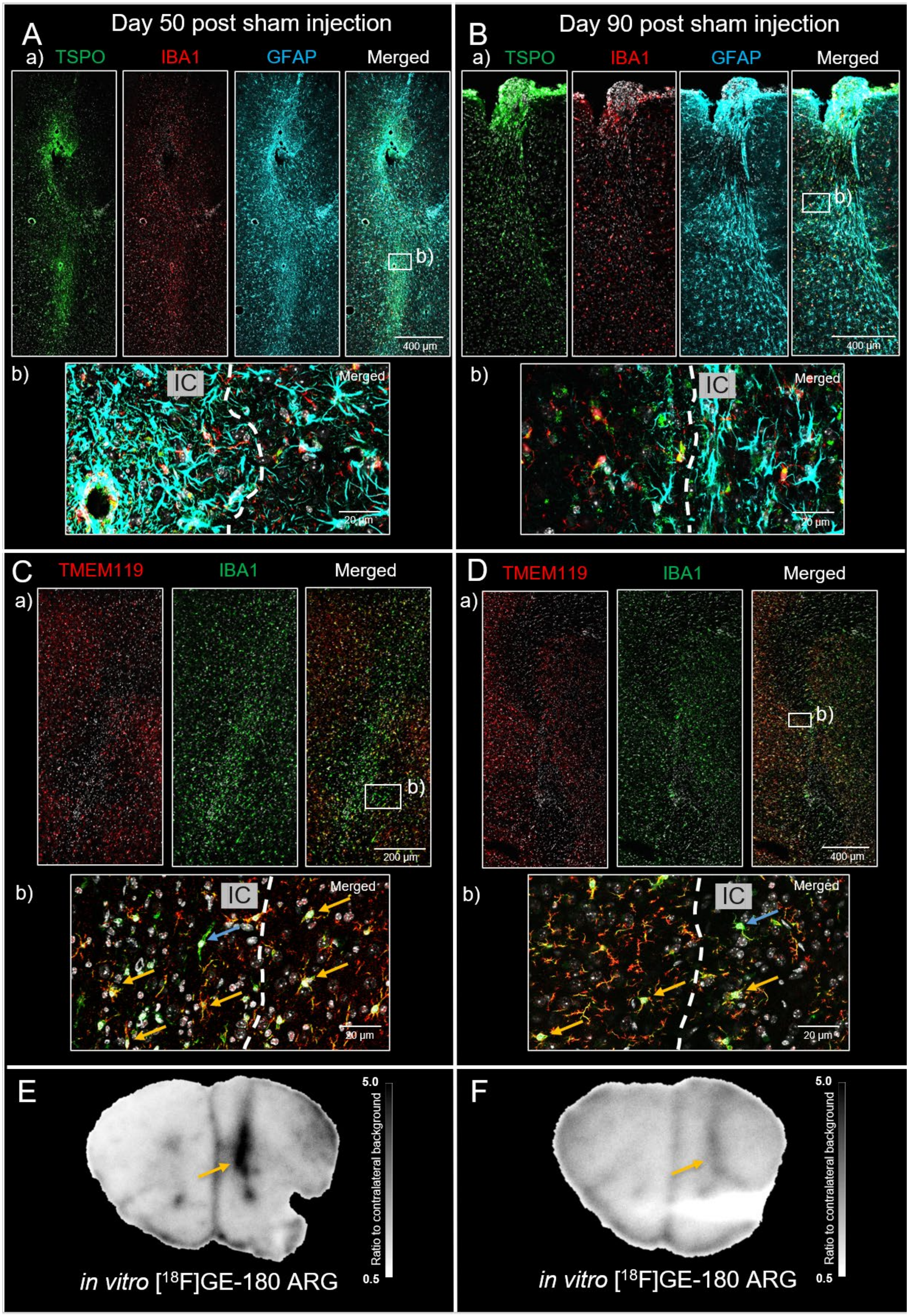

171

172 **Supplementary Figure S3**

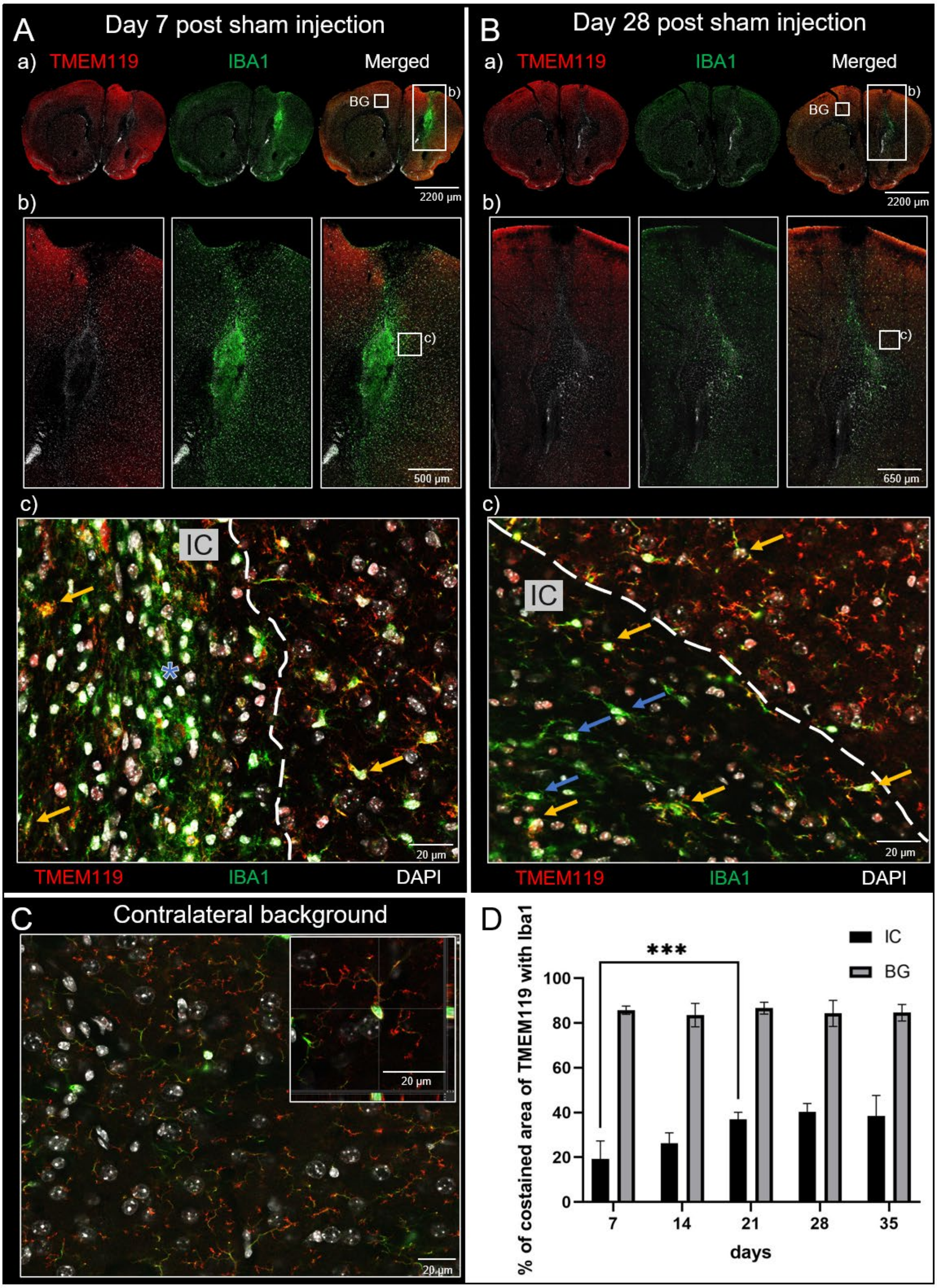

173

174

175

176 **Supplementary Figure S4**

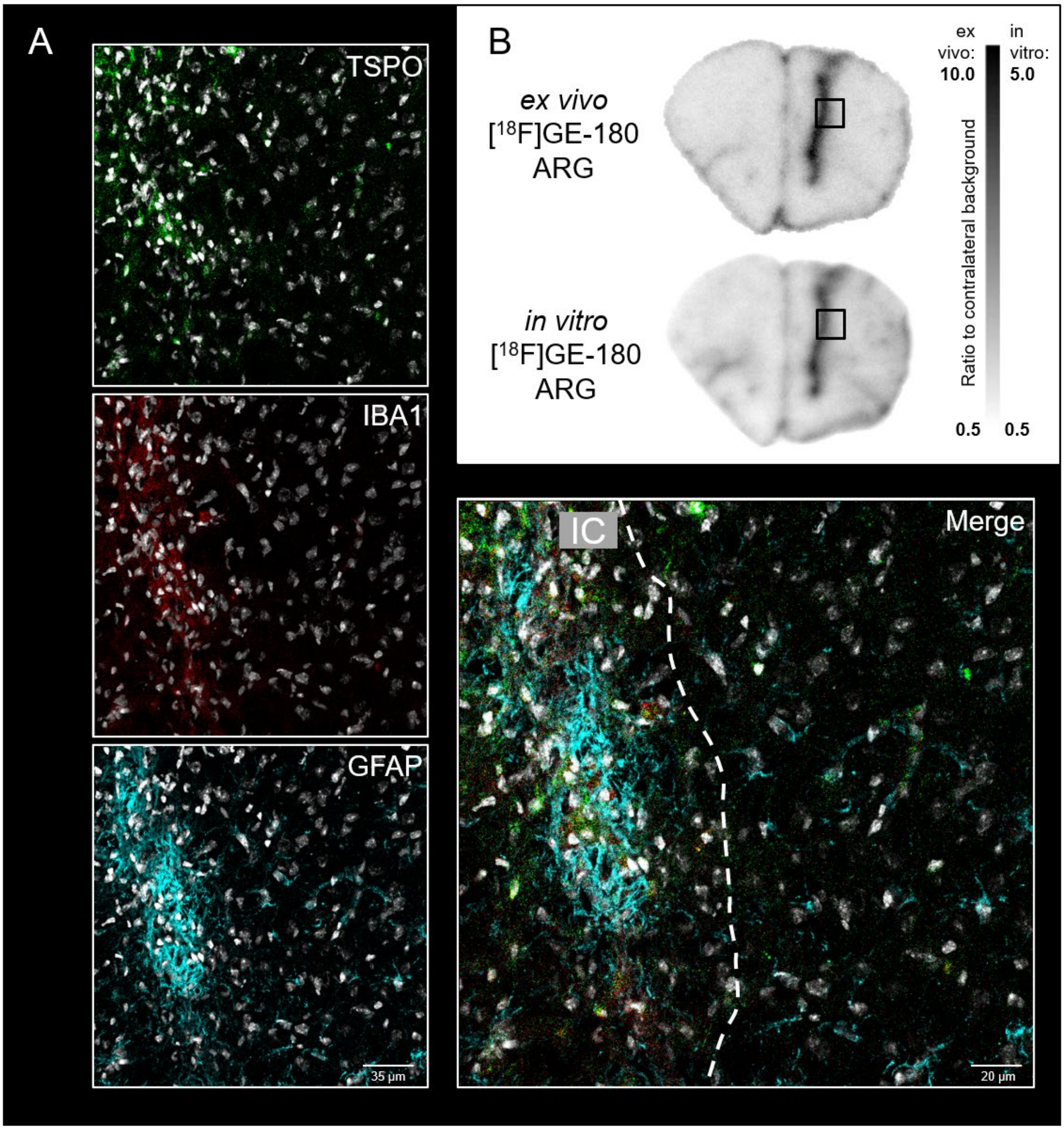

177

178

179

180

181

182

183

184

Supplementary Figure S5

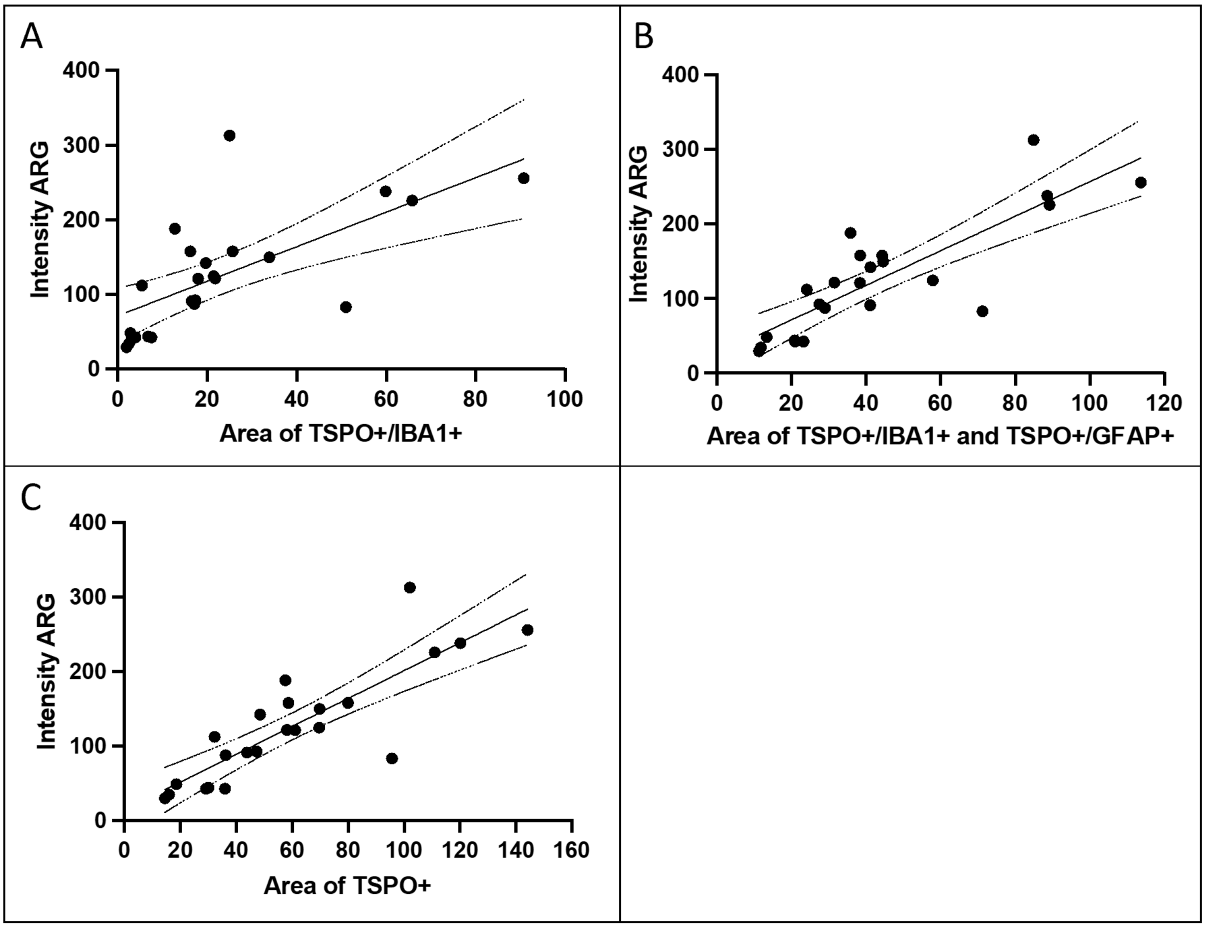

References

1. Holzgreve, A.; Brendel, M.; Gu, S.; Carlsen, J.; Mille, E.; Boning, G.; Mastrella, G.; Unterrainer, M.; Gildehaus, F.J.; Rominger, A., et al. Monitoring of Tumor Growth with [(18)F]-FET PET in a Mouse Model of Glioblastoma: SUV Measurements and Volumetric Approaches. *Front Neurosci* **2016**, *10*, 260, doi:10.3389/fnins.2016.00260.
2. Holzgreve, A.; Pötter, D.; Brendel, M.; Orth, M.; Weidner, L.; Gold, L.; Kirchner, M.A.; Bartos, L.M.; Unterrainer, L.M.; Unterrainer, M., et al. Longitudinal [(18)F]GE-180 PET Imaging Facilitates In Vivo Monitoring of TSPO Expression in the GL261 Glioblastoma Mouse Model. *Biomedicines* **2022**, *10*, doi:10.3390/biomedicines10040738.
3. Deussing, M.; Blume, T.; Vomacka, L.; Mahler, C.; Focke, C.; Todica, A.; Unterrainer, M.; Albert, N.L.; Lindner, S.; von Ungern-Sternberg, B., et al. Coupling between physiological TSPO expression in brain and myocardium allows stabilization of late-phase cerebral [(18)F]GE180 PET quantification. *Neuroimage* **2018**, *165*, 83-91, doi:10.1016/j.neuroimage.2017.10.006.
4. Chen, P.; Hsu, W.H.; Chang, A.; Tan, Z.; Lan, Z.; Zhou, A.; Spring, D.J.; Lang, F.F.; Wang, Y.A.; DePinho, R.A. Circadian Regulator CLOCK Recruits Immune-Suppressive Microglia into the GBM Tumor Microenvironment. *Cancer Discov* **2020**, *10*, 371-381, doi:10.1158/2159-8290.CD-19-0400.
5. Lavisse, S.; Guillermier, M.; Hérard, A.S.; Petit, F.; Delahaye, M.; Van Camp, N.; Ben Haim, L.; Lebon, V.; Remy, P.; Dollé, F., et al. Reactive astrocytes overexpress TSPO and are detected by TSPO positron emission tomography imaging. *J Neurosci* **2012**, *32*, 10809-10818, doi:10.1523/jneurosci.1487-12.2012.
6. Rojas, S.; Martin, A.; Arranz, M.J.; Pareto, D.; Purroy, J.; Verdaguer, E.; Llop, J.; Gomez, V.; Gispert, J.D.; Millan, O., et al. Imaging brain inflammation with [(11)C]PK11195 by PET and induction of the peripheral-type benzodiazepine receptor after transient focal ischemia in rats. *J Cereb Blood Flow Metab* **2007**, *27*, 1975-1986, doi:10.1038/sj.jcbfm.9600500.
7. Wang, Y.; Yue, X.; Kiesewetter, D.O.; Niu, G.; Teng, G.; Chen, X. PET imaging of neuroinflammation in a rat traumatic brain injury model with radiolabeled TSPO ligand DPA-714. *Eur J Nucl Med Mol Imaging* **2014**, *41*, 1440-1449, doi:10.1007/s00259-014-2727-5.
8. Martin, A.; Boisgard, R.; Theze, B.; Van Camp, N.; Kuhnast, B.; Damont, A.; Kassiou, M.; Dolle, F.; Tavitian, B. Evaluation of the PBR/TSPO radioligand [(18)F]DPA-714 in a rat model of focal cerebral ischemia. *J Cereb Blood Flow Metab* **2010**, *30*, 230-241, doi:10.1038/jcbfm.2009.205.
9. Roh, Y.J.; Gong, J.E.; Kim, J.E.; Jin, Y.J.; Song, H.J.; Seol, A.; Park, J.; Lim, Y.; Hwang, D.Y. Comparison of immunophenotypes between Rag2 knockout mice derived from two different sources. *Lab Anim Res* **2023**, *39*, 2, doi:10.1186/s42826-023-00153-8.
10. Shinkai, Y.; Rathbun, G.; Lam, K.P.; Oltz, E.M.; Stewart, V.; Mendelsohn, M.; Charron, J.; Datta, M.; Young, F.; Stall, A.M., et al. RAG-2-deficient mice lack mature lymphocytes owing to inability to initiate V(D)J rearrangement. *Cell* **1992**, *68*, 855-867, doi:10.1016/0092-8674(92)90029-c.
11. Stegen, B.; Nieto, A.; Albrecht, V.; Maas, J.; Orth, M.; Neumaier, K.; Reinhardt, S.; Weick-Kleemann, M.; Goetz, W.; Reinhart, M., et al. Contrast-enhanced, conebeam CT-based, fractionated radiotherapy and follow-up monitoring of orthotopic mouse glioblastoma: a proof-of-concept study. *Radiat Oncol* **2020**, *15*, 19, doi:10.1186/s13014-020-1470-2.
12. Awde, A.R.; Boisgard, R.; Thézé, B.; Dubois, A.; Zheng, J.; Dollé, F.; Jacobs, A.H.; Tavitian, B.; Winkeler, A. The translocator protein radioligand 18F-DPA-714 monitors antitumor effect of erufosine in a rat 9L intracranial glioma model. *J Nucl Med* **2013**, *54*, 2125-2131, doi:10.2967/jnumed.112.118794.
